# Detecting vision problems in children

**Published:** 2024-02-09

**Authors:** P Vijayalakshmi

**Affiliations:** 1 Senior Paediatric Ophthalmologist and Chief of Vision Rehabilitation Center: Aravind Eye Care System, Madurai, India.

**Screening can detect eye conditions early – which is especially important in children, who need good vision in order to develop.**

**Figure:**
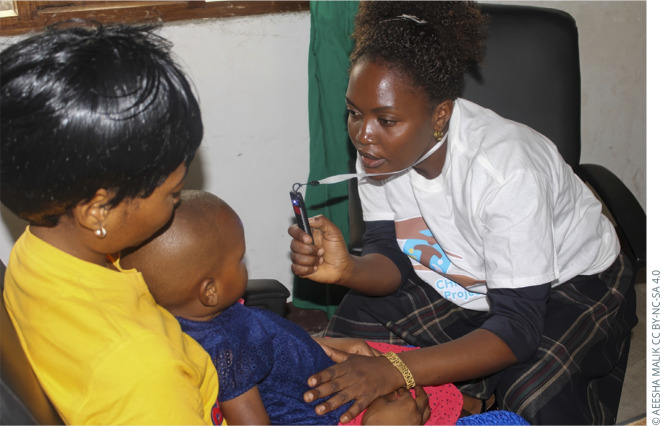
Using the Arclight ophthalmoscope to examine a child's eyes. tanzania

The purpose of screening the eyes of children is to detect specific conditions in as many children in the population as possible, as soon as possible. The types of eye conditions that benefit from screening are those for which early detection and treatment improves visual or health outcomes, and for which there are reliable screening tests that are simple to use, and safe.

Screening on its own is not enough, however. There must also be eye care services where the children who fail screening tests can undergo a comprehensive eye examination to diagnose why they failed the test, and where they can receive effective treatment.

The eye conditions in children that can usefully be screened for are summarised in Table 1.

**Table 1:** Eye conditions which can be screened for in children and adolescents in different age groups

Age	Conditions to screen for	Screening tests and tools	Who can screen
**Preterm neonates**	Retinopathy of prematurity (ROP)	Retinal examination (using an indirect ophthalmoscope Retinal imaging with remote image grading	Ophthalmologist (clinical examination using an indirect ophthalmoscope) Technician or nurse (retinal imaging) followed by grading by an ophthalmologist
**Newborns up to 5 years**	Structural abnormalities of the eyelid Eye size Corneal clarity Squint	Torchlight examination	Trained primary health care worker Pediatrician Experienced health professional
	Lens opacities (cataract) Retinoblastoma	Red reflex test (using an indirect ophthalmoscope or Arclight)	Trained primary health care worker Pediatrician Experienced (eye) health professional
**Children aged 6 years and above (including adolescents)**	Vision impairment Amblyopia and squint	Visual acuity test Torchlight examination	Teachers Health professionals

The screening tests listed in Table 1 may also detect conditions which are not treatable. However, **all** children who fail **any** screening test must be referred for examination by an eye care professional. For example, torchlight examination of a newborn may detect microphthalmos, and fundal (red) reflex testing may detect choroidal coloboma. Neither of these conditions need immediate intervention, but it is important to make the diagnosis.

## Newborn screening

Screening the eyes is now recommended by the World Health Organization as part of the general examination of all newborns.^1^ This can be done by the same person who carries out the general newborn examination, once they have received some additional training.

There are two parts to newborn eye screening:

1. Using a torch to look at the eyelids, to check the size of the eyes and the clarity of the corneas.
2. Eliciting the fundal (red) reflex, which can be done with a direct ophthalmoscope such as the Arclight. Sometimes, the eyelids of newborns are a bit swollen and red reflex testing is difficult; the test can then be delayed until the baby is 6–8 weeks old.

Arclights can easily be attached to a smartphone, which means you can take a video or photographs (Figure 1b). This is useful if you want to discuss what you have seen with somebody else.

Arclights have a small solar panel and batteries are not necessary; they are very light and inexpensive (US $10–15) and come with a lanyard and an otoscope for examining ears. Fundal reflex testing can also be undertaken in young children (and people of any age).

Teaching videos can be found here https://tinyurl.com/CEHJarclight

Screening **preterm babies** for retinopathy of prematurity is covered in detail in an earlier issue of the Journal (http://tinyurl.com/CEHJretinopathy).

## Preschool-age screening

The same screening tests as for newborns can be used for preschool-aged children.

Whether preschool children should also be screened for amblyopia (‘lazy eyes’) is controversial, as this would require measuring their visual acuity, which can be very difficult.

A torchlight examination and fundal reflex testing should detect squint – a common cause of amblyopia. There is also very limited evidence on whether the management of amblyopia due to uncorrected refractive error (such as intermittent patching of the ‘good’ eye before the age of 5 years) has better outcomes than management after the age of 5 years.^2^

Screening preschool-aged children for refractive errors is also controversial, as they are too young to have developed myopia (short sightedness). Hyperopia (longsightedness), if present, resolves spontaneously in most young children.

## Children aged 6 years and above (including adolescents)

The main purpose of screening school-age children is to detect and manage **uncorrected refractive errors**. The most common screening test is a visual acuity measurement during which only one line of the Snellen chart needs to be used – either the 6/9 line or the 6/12 line. Each eye is tested separately.

All children who fail the screening test should undergo refraction by an experienced optometrist, who then also measures the corrected visual acuity. If the vision does not improve, the child needs to be examined to rule out other causes of vision impairment.

If correction does improve the vision, it is important to follow the prescribing guidelines drawn up by IAPB (https://tinyurl.com/ypnja65y). Children whose vision only improves by one line of acuity are very unlikely to wear their spectacles, for example.^3^

Autorefractors and other technology can aid the screening process, but more evidence is needed on their effectiveness.

The age at which vision screening should start is very context-specific and depends on the age at which myopia commonly starts to develop in the child population in that country or region. For example, in China and southeast Asian countries, myopia can start at primary school age, and screening younger children is, therefore, warranted. However, in other parts of the world, myopia doesn't usually start until the age of 9 to 11 years.

School eye health programmes should not focus solely on detecting uncorrected refractive errors, as some children will have other conditions which need treatment, such as infective or allergic conjunctivitis, squint, or cataract.

**Figure:**
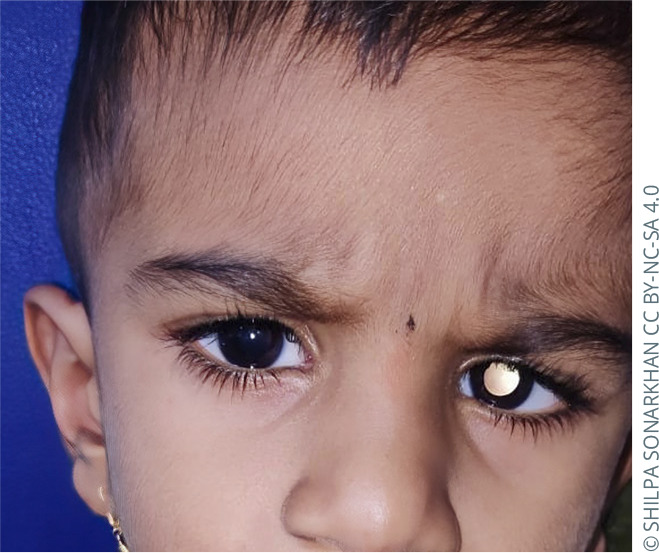
A child with retinoblastoma in the left eye, visible when using a torch light or ophthalmoscope to elicit the red reflex. india

## Summary

Screening, using the range of methods outlined above, can detect eye conditions early. For all age groups of children, it is very important that processes are put in place to ensure that children who fail a screening test are examined by an eye health professional for a diagnosis, and that treatment is provided as soon as possible, whether it is spectacles, cataract surgery, or treatment of retinoblastoma or amblyopia.

**How to elicit the fundal (red) reflex using the Arclight ophthalmoscope**

**Figure 1a:**
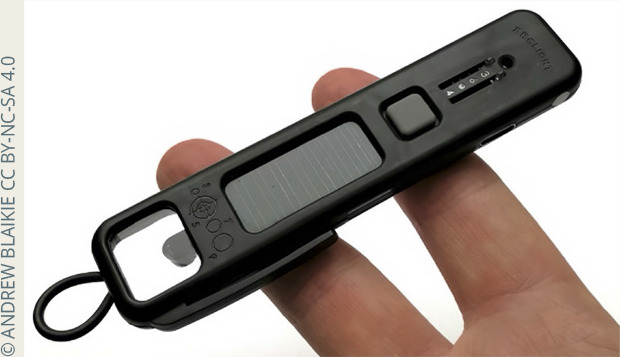
The Arclight ophthalmoscope

**Figure 1b:**
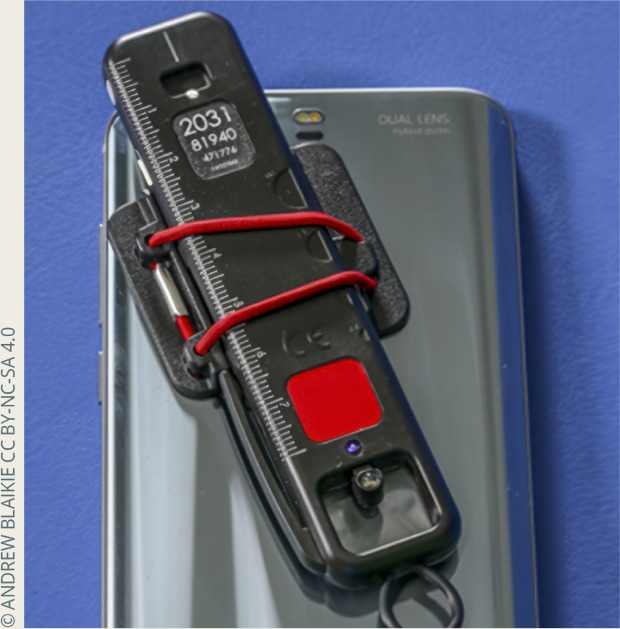
Arclight attached to a smartphone

1. Set the lens of the Arclight or direct ophthalmoscope to 0.
2. Explain to the mother what you are going to do.
3. Position yourself so that you are about a metre away from the baby or child, with your eyes at the same level as the baby's eyes.
4. If the baby's eyes stay closed, ask the carer to hold the baby over their shoulder; this usually makes the baby lift up their head and open their eyes.
5. Shine the Arclight into the baby's eyes so that you can see the reflex in both eyes at the same time. This enables you to compare them.
6. Look carefully at the reflex in both eyes to see whether the reflexes are the same colour and brightness in both eyes. Is there a ‘shadow’ obscuring part or all of the reflex?

**Figure 2:**
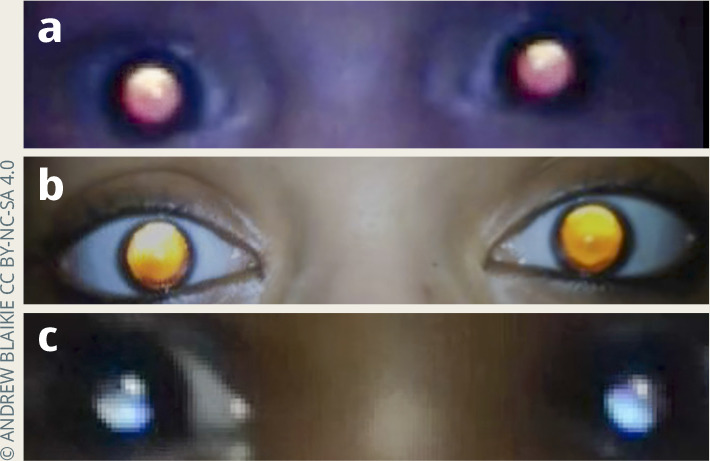
Normal fundal reflex in Caucasian children (**a** and **b**). In African and Asian babies, the normal reflex can have a blueish appearance (**c**).

**Figure 3:**
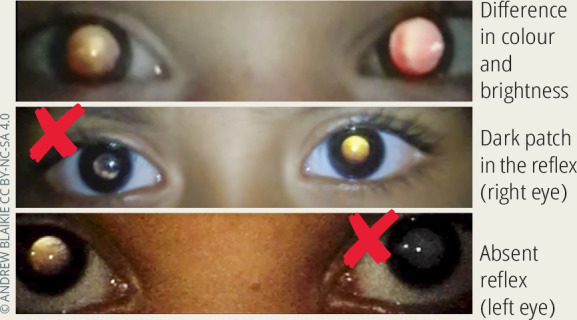
Abnormal fundal reflexes
